# Genetic Interaction Studies Reveal Superior Performance of *Rhizobium tropici* CIAT899 on a Range of Diverse East African Common Bean (Phaseolus vulgaris L.) Genotypes

**DOI:** 10.1128/AEM.01763-19

**Published:** 2019-09-27

**Authors:** A.H. Gunnabo, R. Geurts, E. Wolde-meskel, T. Degefu, K.E. Giller, J. van Heerwaarden

**Affiliations:** 1 Plant Production Systems Group, Wageningen University and Research, The Netherlands; 2 World Agroforestry Centre (ICRAF), Addis Ababa, Ethiopia; 3 International Crops Research Institute for the Semi-Arid Tropics, Addis Ababa, Ethiopia; 4 Laboratory of Molecular Biology, Department of Plant Science, Wageningen University and Research, The Netherlands

**Keywords:** bean genotypes, genotype by strain interaction, N_2_ fixation, nodulation, rhizobium strains

## Abstract

We studied symbiotic performance of factorial combinations of diverse rhizobial genotypes (G_R_) and East African common bean varieties (G_L_) that comprise Andean and Mesoamerican genetic groups. An initial wide screening in modified Leonard Jars (LJ) was followed by evaluation of a subset of strains and genotypes in pots (contained the same, sterile medium) in which fixed nitrogen was also quantified. An additive main effect and multiplicative interaction (AMMI) model was used to identify the contribution of individual strains and plant genotypes to the G_L_ x G_R_ interaction. Strong and highly significant G_L_ x G_R_ interaction was found in the LJ experiment but with little evidence of a relation to genetic background or growth habit. The interaction was much weaker in the pot experiment, with all bean genotypes and *Rhizobium* strains having relatively stable performance. We found that *R. etli* strain CFN42 and *R. tropici* strains CIAT899 and NAK91 were effective across bean genotypes but with the latter showing evidence of positive interaction with two specific bean genotypes. This suggests that selection of bean varieties based on their response to inoculation could be possible. On the other hand, we show that symbiotic performance is not predicted by any *a priori* grouping, limiting the scope for more general recommendations. The fact that the strength and pattern of G_L_ x G_R_ depended on growing conditions provides an important cautionary message for future studies.

## Importance

The existence of genotype by strain (G_L_ x G_R_) interaction has implications for the expected stability of performance of legume inoculants and could represent both challenges and opportunities for improvement of nitrogen fixation. We find that significant genotype by strain interaction exists in common bean *(Phaseolus vulgaris L.)* but that the strength and direction of this interaction depends on the growing environment used to evaluate biomass. Strong genotype and strain main effects, combined with a lack of predictable patterns in (G_L_ x G_R_), suggests that at best individual bean genotypes and strains may be selected for superior additive performance. The observation that the screening environment may affect experimental outcome of G_L_ x G_R_ means that identified patterns should be corroborated under more realistic conditions.

## Introduction

Common bean (*Phaseolus vulgaris* L.) is a globally important grain legume (Fabaceae) that originated from the New World. It has two main diversification centres: Mesoamerica (from Mexico to the Northern region of South America) and the Andes (from Southern Peru to the North of Argentina) (1), giving rise to two distinct gene pools, “Andean” and “Mesoamerican”, which each contain varieties with different growth habits such as determinate bush (B), bush indeterminate (BI), prostrate indeterminate (PI) and prostrate climbing (2–4). Varieties from both gene pools have been distributed throughout the world and are major food crops in Eastern and southern Africa.

It is widely thought that common bean (hereafter referred to as “bean”) has low N_2_-fixation potential compared to other legumes (5–7) but reports of strong positive responses to inoculation (6–10) suggests that this may be overcome by providing highly effective rhizobia in abundance. Results from inoculation studies have been mixed however (11–14) and there is currently no consensus as to what causes this variation. One possible factor could be that the numerous indigenous rhizobia commonly found in soils where bean is grown (5) limit inoculation response by outcompeting the elite strain. Although some studies seem to confirm this (15, 16) others have observed strong responses in soils with very high rhizobial population densities (17, 18). The latter may suggest that that symbiotic effectiveness, of either the inoculant or the local population, rather than abundance of indigenous rhizobia could be an issue. The benefits of inoculant may be compromised if the elite strain is outcompeted by less effective native rhizobia, although a high inoculum titre is expected to help to overcome this limitation (15, 19). Providing that the inoculant strain is present in sufficient numbers, differences in effectiveness may still occur due to genetic factors related to the rhizobia in the root zone, to the host plant genotype or to the interaction between the two (9, 20–23). Here, we consider all three aspects as potential determinants of inoculation success.

With respect to the effect of host genotype, studies in both natural and cultivated legumes have shown genetic differences in the preference for specific symbionts (24) as well as in the additional biomass accumulated as a result of symbiosis (10, 14, 19, 25). Such differences are possibly due to large genetic differences observed in symbionts and also to the co-adaptation of cultivar and bacteria (7). In beans, genotype specific variation for nodulation (infectiveness) and symbiotic effectiveness has been found (26). At a higher level, Andean and Mesoamerican genotypes were reported to show differences in nitrogen fixation under specific conditions (27) while differences between climbing beans and bush beans were also observed (28). It is therefore not unlikely that areas hosting a diversity of bean varieties can show variable symbiotic performance due to genotype. Such is the case of East Africa, which is home to a large diversity of common bean landraces of different genetic origins and growth habits (2, 29) hosting a taxonomically wide range of associated rhizobia (30–32).

As for the role of rhizobial diversity, there is ample evidence that plant-associated symbionts may vary from highly beneficial (symbiotically effective strains) to entirely ineffective (33–35). As a promiscuous host legume (5, 36), bean is able to be infected by a large number of rhizobial species (20, 37, 38) so the potential for differential symbiotic outcomes is evident. Although mechanisms for discriminating against ineffective rhizobia, either prior to (39) or after nodulation (40, 41) have been shown to exist in different legumes, it is not known if bean has similar abilities. But, higher nodulation performance by *R. etli* strains containing a nodC type-α than the strains that contain a nodC type-δ in Mesoamerican beans is considered as a good example of prior selection of strains for nodulation in bean plants (20, 42, 43). More than 27 species of rhizobia have been isolated from bean (32, 44–48), among which *Rhizobium tropici* (49), *R. etli* (50), *R. phaseoli* (51), *R. giardinii* and *R. gallicum* (52) are most commonly mentioned. *R. tropici* and *R. etli* predominate in central American soils (7, 20) and the type strain *R. tropici* CIAT899 (isolated from Colombia) is commonly used as a commercial inoculants in Latin America (19, 53) and Africa (30, 32, 54, 55). In addition to *Rhizobium* spp. bean also associates with other rhizobia such as *Sinorhizobium meliloti, S. fredii* and *S. americanum* (56, 57), *S. arboris* and *S. kostiense* (7), *Bradyrhizobium* spp. (58, 59), and the P-proteobacteria *Cupriavidus necator* (60), *Burkholderia phymatum* (61) and *Paraburkholderia nodosa* (62). Significant variation in symbiotic effectiveness in bean has been demonstrated for strains from several of the above species (7). This means that there is scope for identifying rhizobial strains of superior effectiveness, while on the other hand association with competitive but ineffective strains from the background population may be part of the reason that response to inoculation in bean has been found to be erratic (6, 7, 9).

Apart from genetic differences between legume and rhizobium genotypes, the interaction between both factors, so called genotype (G_L_) x rhizobium genotype (G_R_) interaction (G_L_ x G_R_), is of particular interest as a determinant of inoculation success. G_L_ x G_R_ interaction is the phenomenon whereby the symbiotic performance of specific combinations of strains and bean genotypes is significantly better or worse than expected based on their respective average performance (22, 63). This dependence of relative superiority of a rhizobium strain on host genotype has been proposed as an evolutionary driver of the maintenance of genetic variation in rhizobial effectiveness (i.e. the partner mismatch hypothesis 15, 18, 53). The existence of a G_L_ x G_R_ interaction is well accepted in different legumes (67–72) including bean, although evidence for the latter has been mixed from no effect (12) to a highly significant interaction (21, 22, 73). Where interaction was observed, it could either be due to differential nodulation (nod+/nod-) (20), fixation (fix+/fix-) (74) and biomass phenotypes (22, 75–78).

At a practical level, the occurrence of G_L_ x G_R_ interaction can represent both challenges and opportunities for the development of effective inoculants. If the outcome of the interaction is unpredictable, G_L_ x G_R_ interaction can pose problems for the development of stably performing inoculant, since some legume varieties may not combine well with the elite strain. If, on the other hand, the interaction is predictable based on the taxonomy or genetic origin of either rhizobia or legume genotypes due to coevolution, it could allow for the targeting of inoculant strains to types of varieties. This is, in a way, analogous to the targeting of plant varieties to environments as is common in plant breeding (79).

Although some authors argue that coevolution is not likely to occur in the legume-rhizobium symbiosis, there is strong evidence that symbiotic bacteria do co-evolve with their hosts in the centres of legume origin and diversification (for a review see 80, 81). This could be targeted for better host-strain combinations although coevolution is not guaranteed to result in the most effective combinations (41). Lie et al. (82) presents several examples in pea, where compatible strains were restricted to the same regions as their host genotypes. In bean, there is some reason to expect predictable patterns of G_L_ x G_R_ interaction. Co-inoculation studies showed that varieties from Andean and Mesoamerican genepools showed clear nodulation preference for strains of *R. etli* typical of these respective regions (20) for example. Less likely, but worth considering are possible specific associations due to growth habit, which is known to be linked to growing environment (4), or country of varietal origin.

Thus far, studies into G_L_ x G_R_ in bean have been conducted using a limited genetic range of genotypes and strains, and none thus far has evaluated the effects of domestication genepool on the relative symbiotic performance of diverse rhizobia. In this study, we quantified interaction effects in symbiotic effectiveness in a set of reference and native rhizobial strains and East African bean landraces specifically selected to represent the genetic diversity among rhizobium strains and bean accessions. We thereby test for the contribution of genepool, growth habit and country of origin to the interaction performance. Using statistical techniques commonly used in genotype by environment studies, we evaluate patterns of interaction to determine if universally stable or specifically superior combinations of rhizobia and bean varieties can be identified.

## Materials and Methods

### Selection of bean genotypes and rhizobium strains

The choice of bean genotypes was made from a total of 192 accessions collected from a range of common bean production ecologies in Ethiopia and Kenya, and previously characterized genetically by simple sequence repeat (SSR) markers (2). The 192 accessions were assigned to 18 genetic groups using ward clustering on the Euclidean distance matrix along the first 17 principal components calculated from the matrix containing the 0, 1 or 2 scores for each marker allele (83). The genetic distance between groups was calculated as the pairwise fixation coefficient (84). A neighbour joining tree (85) was reconstructed to visualise the relationships between genetic groups (Fig. S1). We selected 10 genotypes, ensuring equal representation of Andean and Mesoamerican genepools, growth habit and country of origin. The selected genotypes were obtained from the Centre for International Agriculture in Tropics (CIAT), Colombia, and subsequently propagated in the greenhouse. The passport information for the genotypes used (Table 1) was modified from CIAT documentation and Asfaw et al. (2).

**Table 1:** Bean genotypes used in G_L_ x G_R_ experiments

Accession number	100 Seed weight (g)	Growth habit	Use	Country of collection	Genetic cluster	Genepoo 1	Tested in pot
G764	47.8	C = IV	Snap Bean	Ethiopia	5	Andean	No
G1372	34.6	B = I	Dry Bean	Kenya	4	Andean	Yes
G11481	44.3	B = I	Dry Bean	Ethiopia	3	Andean	No
G20528	61.7	B = I	Dry Bean	Kenya	14	Andean	No
G20544	53.1	B = I	Dry Bean	Kenya	15	Andean	Yes
G2889A	19	IB = II	Dry Bean	Kenya	2	Meso	No
G20141	21.6	IP= III	Dry Bean	Ethiopia	8	Meso	Yes
G20142	23.9	IP = III	Dry Bean	Ethiopia	11	Meso	Yes
G24484	28	C = IV	Dry Bean	Kenya	13	Meso	Yes
G50545	26.3	IP = III	Dry Bean	Kenya	17	Meso	No

Where: Meso = Mesoamerican genepool, and Andean = the Andean genepool; C = Indeterminate climbing; B = Determinate bush; IB = Indeterminate bush;

IP = Indeterminate prostrate

Eight rhizobial strains were selected to study their symbiotic performance in combination with the selected bean genotypes. The selection consisted of five type strains (as representative genotypes of *Rhizobium* species for which genetic information is available), four of which represented species reported to nodulate bean, a Kenyan strain and two newly collected Ethiopian strains (Table 2). The type strains were chosen to represent the dominant taxonomic groups found to occupy bean root nodules such as *R. phaseoli, R. etli, R. tropici* and a strain from the genus *Sinorhizobium, S. meliloti* (49–51, 57, 86, 87) and were imported from the Laboratory of Microbiology, University of Gent rhizobial collection centre (LMG), Belgium. The newly isolated local strains were selected based on site of isolation and authentication tests, since at the time local strains had not been characterized. Based on the effectiveness test results, local strains were phylogenetically characterized for symbiotic gene (*nodC*) and concatenated housekeeping (core) gene (16s rRNA, *glnII, gyrB* and *recA*) sequences.

**Table 2:** Rhizobium strains used in G_L_ x G_R_ experiments

Strains code	Species	Host pant	Geographic origin	References	Tested in pot
CFN 42	*R. etli*	*P. vulgaris*	Mexico	(1)	Yes
CIAT 899	*R. tropici*	*P. vulgaris*	Colombia	(2)	Yes
ATCC 14482	*R. phaseoli*	*P. vulgaris*	Beltsville, USA	(3)	Yes
NAE136	*Rhizobium* sp.	*P. vulgaris*	Hadiya, Ethiopia	this work	Yes
NAE182	*Rhizobium* sp.	*P. vulgaris*	Borena, Ethiopia	this work	Yes
LMG 6133	*S. meliloti*	*Medicago sativa*	Virginia, USA	(4)	No
LMG 23946	*R. multihospitium*	*H. halodendron*	Xinjiang, China	(5)	No
NAK91	*Rhizobium* sp.	*P. vulgaris*	Kenya	N_2_Africa-Kenya	Yes

Where: NAE = N_2_Africa-Ethiopia; LMG = Laboratory of Microbiology, University of Gent rhizobial collection centre, NAK = N_2_Africa-Kenya, *H* = *Halimodendron; P* = *Phaseolus; R* = *Rhizobium; S* = *Sinorhizobium*

IP = Indeterminate prostrate

### Molecular characterization of local rhizobial isolates

The selected local rhizobial strains were initially trapped from soils collected from Hadiya (Lat. 7^O^ 40’3 9”, Long. 38^O^ 14’ 45”, Altitude 2030 m.a.s.l. and soil pH 7.45) and Borena (Lat. 5^O^54’ 47”, Long. 38 ^O^ 9’ 46”, Altitude 1691.57 m.a.s.l. and soil pH 6.61) in southern Ethiopia. “Nasir” local bean variety was used to trap the strains from the soils in a screenhouse at Hawassa College of Agriculture. The strains were then isolated from the root nodules of the bean varieties according to procedures described previously (88).

A rhizobial colony growing on peptone-salts-yeast extract (PSY) medium was picked and diluted in 50 ul MQ (Milli-Q or ‘ultrapure’) water for 10 minutes. A 2ul aliquot of the colony suspension was used to amplify housekeeping genes (16S rRNA, *glnII, recA* and *gyrB)* and symbiotic target gene (*nodC).* Primers and PCR amplification conditions used for each locus are listed in (Table 3). For all the PCR reactions, PCR master mix was prepared by 17.4ul MQ water, 2.5ul (10x) Dream Taq buffer, 1ul (10mM/ul each forward and reverse primers) and 0.1ul (5U/ul) Dream Taq DNA polymerase enzyme (Thermo Fisher Scientific Inc.) to make final reaction volume of 25ul. PCR products were cleaned using Thermo-scientific PCR product cleaning kit and sequenced by Macrogen Inc. (The Netherlands). The GenBank accession numbers of sequences determined in this work are MK251991, MK252267 and MK2553259 for the 16S rRNA; MN453339, MN453340 and MN453341 for *nodC;* MN453342, MN453343 and MN453344 for *nifH;* MN453345, MN453346 and MN453347 for *glnII;* MN453348, MN453349 and MN453350 for *gyrB;* and MN453351; MN453352 and MN453353 for *recA.*

**Table 3:** List of primers and their PCR conditions

Loci	Primer and their target gene position	Primer sequence 5’-3’	PCR condition	References
*16S rRNA*	63F	CAG GCC TAA CAC ATG CAA GTC	5’ 95^0^C, 35x(0.30’ 95^0^C, 0.30’ 55^0^C, 1’ 72^0^C), 7’ 72^0^C	(91)
	1389R	ACG GGC GGT GTG TAC AAG		
*nodC*	nodCfor540 (544-566)	TGA TYG AYA TGG ART AYT GGC T	2’ 98°C, 34x(0.15’98°C, 20’ 63°C, 0.20’ 72°C), 5’72°C	(31)
	nodCrev1160 (1164-1184)	CGY GAC ARC CAR TCG CTR TTG		
*nifH*	nifH-1F (367-389)	GTC TCC TAT GAC GTG CTC GG	5’95°C, 35x(0.30’ 95°C, 0.30’57 °C, 1.’ 2°C), 7’72 °C	(31)
	nifH-1R (794-774)	GCT TCC ATG GTG ATC GGG GT		
*recA*	recA-6F (16–31)	CGK CTS GTA GAG GAY AAA TCG GTG GA	10’ 95^0^C, 35x(0.30’ 95^0^C, 0.45’ 57^0^C, 1’ 72^0^C), 7’ 72^0^C	(31)
	recA-555R (555-530)	CGR ATC TGG TTG ATG AAG ATC ACCAT		
*rpoB*	rpoB-83F (83–103)	CCT SAT CGA GGT TCA CAG AAG GC	5’ 95^0^C, 3x(2’ 94^0^C, 2’ 58^0^C, 1’ 72^0^C), 30x(0.30’ 94^0^C,1’ 58^0^C, 1’ 72^0^C ), 5’ 72^0^C	(31)
	rpoB-1061R (1081-1061)	AGC GTG TTG CGG ATA TAG GCG		
*glnll*	glnII-12F	YAA GCT CGA CTA CAT YTC	10’ 95^0^C, 35x(0.30’ 95^0^C, 0.45’	
	glnII-689R	TGC ATG CCS GAG CCG TTC CA	57^0^C, l’ 72^0^C), 7’ 72^0^C	
gyrB	gyrB343F	TTC GAC CAG AAY TCC TAY AAG G	5’ 95^0^C, 3x(2’ 94^0^C, 2’ 58^0^C, 1’ 72^0^C), 30x(0.30’ 94^0^C,1’ 58^0^C, 1’ 72^0^C ), 5’ 72^0^C	(92)
	gyrB1043R	AGC TTG TCC TTS GTC TGC G		

DNA sequence data were trimmed using SnapGene Viewer software (GSL Biotech, Chicago, IL). The edited sequences were compared to GenBank (https://blast.ncbi.nlm.nih.gov/). Multiple nucleotide sequence alignments were carried out using CLUSTALW (89) in MEGA7 software (90). Phylogenetic trees of each and concatenated loci were constructed using the maximum likelihood method based on the General Time Reversible (GTR) model and evolutionary rate differences among sites was modelled by Gamma distribution (+G, parameter); positions with gaps in any sequence were discarded. The robustness of the tree topology was calculated from bootstrap analysis with 1000 replications. The percentage similarity of the genes was estimated using the Kimura 2-parameter distance matrix correction model as implemented in MEGA version 7 (90).

### Experimental design: G_L_ x G_R_ interaction in Leonard Jars and pots

Symbiotic interaction was studied using factorial combinations of the selected bean genotypes and rhizobial strains in 0.7 litre modified Leonard jars and 4 litre capacity pots (91, 92). Jars were used to evaluate all possible genotype by strain combinations for nodulation (Nod+/-), fixation (Fix+/-) and biomass. Pots were used to confirm the symbiotic performance of all combinations of a subset of genotypes and strains that were consistently Nod+ and Fix+ in the Jar experiment. In this experiment, nitrogen content was determined in addition to nodulation, fixation and biomass. Since pots had a larger rooting volume, conditions for growth and N_2_ fixation were assumed to be more representative of those in the field.

The modified Leonard jars were prepared following standard procedures (91). River sand, pre-treated with concentrated sulphuric acid, was washed several times in tap water to neutralise the effect of the acid and then air dried, added into the jars, covered with aluminium foil and autoclaved at 121^O^C for 15 minutes at 67 kg/m/s^2^ pressure. The entire jars were covered with aluminium foil to minimise the high temperatures in the screen-house. Pot preparation was adopted from (92) by which 30 cm top width x 20 cm depth pots were wrapped in aluminium foil and autoclaved. One-inch PVC tubes were cut into 30 cm lengths; the tubes were wrapped in aluminium foil and autoclaved separately. River sand was prepared as described above and autoclaved independently in plastic bags. The autoclaved sand was aseptically transferred into the pots containing sterile PVC tube in a laminar flow hood and covered with aluminium foil until used.

Seeds of the selected bean genotypes were surface sterilised by rinsing in 96% ethanol for 8-30 seconds and then in 4% sodium hypochlorite for 4 minutes. They were then cleaned in six changes of sterile distilled water. The seeds were pre-germinated in sterilised Petri dishes on sterile tissue paper. The pre-germinated seeds were aseptically transplanted into the jars (92).

The selected rhizobial strains were grown in yeast extract mannitol broth (YMB) in a rotary shaker at 130 revolutions per minute (91, 92). The rhizobial broth culture at their logarithmic growth phase (~10^9^ cells ml^-1^) was inoculated (1 ml) to the base of the seedlings growing in the jars. Ten positive (uninoculated but N-fertilized) and ten negative (uninoculated and unfertilized) controls were included to get 500 experimental units for jar and 200 total experimental units for pot and arranged in a completely randomized block design (RCBD). Each of the treatment combinations and controls were replicated five times and supplemented with Jensen’s N-free nutrient solution once in every week and watered with sterilised distilled water as needed. Positive controls were additionally supplied with 300 ml quarter strength of 0.5 g/L KNO_3_ (of which N is 0.06927 g/L). After growing for 45 days in the screening house, the plants were carefully uprooted, roots washed with tap water and assessed for nodulation and N_2_ fixation phenotypes.

The presence or absence of nodules was recorded as “nod+” or “nod-”, respectively. Nodules were carefully detached from the roots and cut into sections to examine their internal colour. Nodules with pink or red internal colours were recorded as “fix+”, which indicates the presence of leghaemoglobin and effective symbiotic N_2_ fixation and small nodules with green or white internal colours were recorded as “fix-”, indicating ineffective nodules. Thus, each jar was scored as fully effective (nod+/fix+), ineffective (nod+/fix-) and not nodulating (nod/fix-). Furthermore, nodule number per plant was counted, nodules were separated from the roots, dried and weighed.

Subsequently, five bean genotypes and six rhizobial strains with effective, ineffective and minimal inconsistencies in terms of nod(+/-) and fix(+/-) phenotypic response combinations were screened for further pot experiment (Table 1 and 2) in order to confirm their symbiotic interaction and evaluate the extent to which they fix atmospheric N_2_. Thus, the selected seeds were surface sterilised, pre-germinated and aseptically transplanted into pots through small holes in the aluminium foil as described above. Similarly, rhizobial broth culture at their logarithmic growth phase was also inoculated (1 ml) to the base of the seedlings growing in the pots. All the treatment combinations and controls were given Jensen’s N-free nutrient solution and sterilised distilled water through the PVC tubes inserted into the pots (91, 92). The positive controls additionally received KNO_3_ in 5ml of 10 g/L KNO_3_ (of which N is 0.140067 g/L) through the pipes once in every week (92). When the seedlings had established the aluminium cover was carefully removed and replaced by sterile cotton wool to protect dust particles landing on the sand. They were grown for 45 days in the screen house and then assessed for nodulation and N_2_ fixation.

### N concentration in shoots of plants from pot experiment

Nitrogen concentration in plant shoots was estimated using near-infrared spectroscopy (NIRS) method at Nutrition Lab at International Livestock Research Institute (ILRI), Ethiopia, following the standard protocols (93, 94). Plant shoot samples were oven dried at 70^O^C for 48 hours and powdered using mortar and pestle to pass through a 1 mm mesh. The mortar and pestle were cleaned with ethanol after each sample. The prepared samples were again oven dried at 40 ^O^C for overnight and stored in a desiccator containing a silica gel before scanning the samples using an automated NIRS spectrometer. Ten percent of the samples were purposely selected by considering all the genotypes and subjected to Kjeldahl wet-chemical analysis method to determine plant total nitrogen and used to calibrate the NIRS method (93, 94).

The N derived from the atmosphere (Ndfa) was estimated by using N difference method as previously described elsewhere (6, 92, 93). N difference compares total N of the N_2_-fixing legume with that of the negative controls. In order to avoid variation in N_2_ fixation due to bean genotype’s seed differences, Ndfa was calculated separately for each genotype.

$$\text{Total shoot N}\left({\text{TSN}}_{g}\right)=\frac{{SDW}_{g}\times \%\text{N}}{100}$$

$${\text{Ndfa}}_{\left(\text{gm}\right)}={TSN}_{in}-{TSN}_{neg}$$

Where: *SDW*_*g*_ = shoot dry weight in grams; %*N* = a percentage of nitrogen estimated from samples of SDW using NRIS spectroscopy; *TSN_in_* = total shoot nitrogen of inoculated plants; and *TSN_neg_* = mean shoot nitrogen of negative control plants.

Relative amount of fixed nitrogen (RNdfa) was also calculated by dividing Ndfa by total shoot N of the positive control (N-fertilized) plants. Mathematically:

$$\text{RNdfa}=\frac{Ndfa}{{TSN}_{pos}}$$

Where, *TSN_pos_* is the shoot N content of the N-fertilized plants

### Relative symbiotic efficiency (RSE)

The bean genotypes we used have a significant variation in seed sizes and weights as well as in inherited growth performance. Hence, RSE was calculated to determine the relative performance of strains across genotypes. RSE was calculated as:

$$\text{RSE}=\frac{{SDW}_{g_{i}}-{SDW}_{g_{c}}}{{SDW}_{g_{N}}}$$

Where; ${\text{SDW}}_{g_{i}}$. is the shoot dry weight (in gram) of inoculated plants, ${\text{SDW}}_{g_{c}}$ is the shoot dry weight of negative control plants and ${\text{SDW}}_{g_{N}}$ is the yield of N-fertilized positive controls.

This definition is a modification of the one used Brockwell et al. (95) in which the denominator was ${\text{SDW}}_{g_{N}}-{\text{SDW}}_{g_{c}}$. The original denominator may approach zero in individual replicates, e.g. due to experimental variability, which we found to result in a variable with a very skewed distribution, complicating statistical analysis. This redefinition means that the expectation of RSE is now proportional to *N fixed/(N fertiliser+ N seed),* as opposed to *N fixed/N fertiliser* under the original definition. The difference with respect to the original metric should be minor as long as *N fertiliser* > *N seed* and will in any case not affect the relative performance of- or interaction with specific strains.

### Analysis of nodulation, fixation and biomass

The nodulation (nod+, nod-) and fixation (fix+, fix-) phenotypic observation scores for specific genotype-strain combinations were summed for each replication and means of each count returns were predicted for the combinations. The means were then scaled based on minimum and maximum counts of the observation scales per combination to plot and see the patterns of observed phenotypic scores. Finally, G_L_ x G_R_ matrices of mean scores of (nod+/-) and fixation (fix+/-) were visualised by heat-maps in R.

### Quantitative analysis of G_L_ x G_R_ interaction

The effects of genotype, strains and their interaction on the quantitative variables relative shoot dry weight (RSDW) and nitrogen derived from atmosphere (Ndfa), were estimated by fitting the following linear mixed model (*Imm):*

$$Y=\mathrm{Genotype}+\mathrm{Strain}+\mathrm{Genotype}.\mathrm{Strain}+\underset{¯}{\mathrm{Rep}}+\underset{¯}{e}$$

Where *Y* is the response variable as determined by the main effects of *Genotype* and *Strain* and their interaction. *Rep* and *e* are a random replicate effect and residual error (random terms are indicated by underlining), respectively. Significance of fixed effects was tested by a type I ANOVA with Satterthwaite’s approximation to the degrees of freedom as implemented in *ImerTest* package in R version 3.5.0. Model means for each G_L_ x G_R_ combination were calculated with the *predictmeans* function (package *predictmeans).*

Effects of groups of genotypes were modelled as:

$$Y=\mathrm{Group}+\mathrm{Strain}+\mathrm{Genotype}.\mathrm{Strain}+\underset{¯}{\mathrm{Genotype}}+\underset{¯}{\mathrm{Genotype}.\mathrm{Strain}}+\underset{¯}{\mathrm{Rep}}+\underset{¯}{e}$$

Where the random terms *Genotype* and *Strain. Genotype* are the genotype main effect and the genotype times strain interaction.

### Additive main effect and multiplicative interaction (AMMI) model

After establishing the existence of G_L_ x G_R_ interaction an additive main effect and multiplication interaction (AMMI) model was used to decompose the G_L_ x G_R_ interaction. AMMI is a technique that is used widely in the plant breeding literature for the analysis and interpretation of genotype by environment interactions (79, 96, 97). Its main purpose is to reduce the multidimensional patterns of interaction into a small number (typically two) of components that contain as much information on the interaction as possible. This is achieved by fitting a statistical model that first subtracts the G and E main effects before applying singular value decomposition (SVD) of the G x E interaction effects (79, 97). The final model describing the genotype by environment data then becomes (98):

$${\text{Y}}_{\text{ij}}+\mu +\alpha_{i}+\beta \text{j}+\sum_{k=1}^{t}\lambda_{k}\xi_{ik}\eta_{kj}+\epsilon_{ij}$$

Where μ is the overall mean; *α_t_* is the genotype main effect; β_j_ is the environment main effect; *t* is the number of SVD axes retained in the model; *λ_k_* is the singular value for the SVD axis *k*; *ξ*_*ik*_ is the singular value of the genotype i for the SVD axis *k*; *η_kj_* is the singular value of the environment j for the SVD axis k; and *ɛ_tj_* is the error term. By retaining only a subset, *t*, of the components the dimensionality of the interaction is reduced considerably, allowing a description of broader patterns. For *t* = 2, a two-dimensional biplot can be constructed in which environments in which the same genotypes have the same relatively performance (with respect to their mean) will be drawn as vectors pointing in the same direction while the projection of individual genotypes on these vectors will show with which environments, they have a positive or negative interaction. Here, we defined legumes genotypes as environments and strains as genotypes to describe the patterns of G_L_ x G_R_ in terms of N_2_-fixation and relative biomass. AMMI analysis was performed using the *“agricolae“* package in R.

### Phylogenetic relationships of *Rhizobium* strains used in G_L_ x G_R_ interaction

**Fig. 1:**
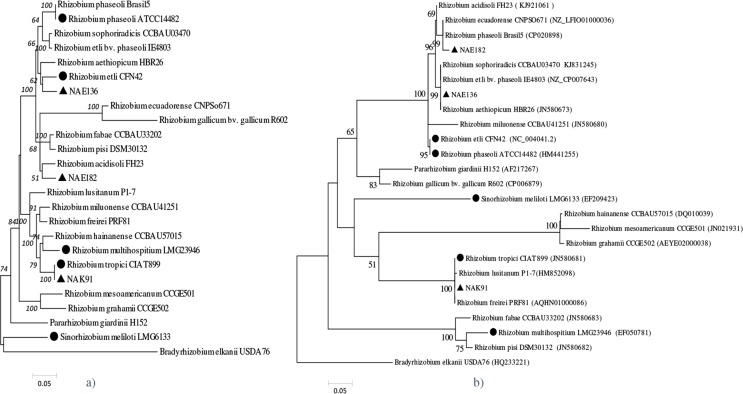
Phylogenetic relation of bean compatible rhizobium species. The evolutionary history was inferred by using the maximum likelihood method based on the General Time Reversible (GTR) model and evolutionary rate differences among sites was modelled by Gamma distribution (+G, parameter). The reconstruction of the phylogenies was based on (a) concatenated gene sequences of 16S rRNA, *glnll, gyrB* and *reck,* (b) the common nodulation gene *nodC.* Strains marked with ▲ are local strains while strains marked with • are the type strains included in the G_L_ x G_R_ study.

For the genetic interaction studies, we aimed to use a diverse range of compatible rhizobium strains and bean genotypes. The phylogenetic analysis of the rhizobium strains on the basis of four concatenated housekeeping genes (16S rRNA, *glnII, recA* and *rpoB)* confirmed the genetic diversity of the selected strains (*R. etli* CNF42, *R. tropici* CIAT899, *R. phaseoli* ATCC14482, *R. multihospitium* LMG23946, and *S. meliloti* LMG6133) and revealed that the three East-African strains (*Rhizobium* sp. NAE136, NAE182 and NAK91) each fell into distinct genetic clusters (Fig. 1a). The East-African strains are all of the *Rhizobium* genus, with a strain NAE136 representing a distinct phylogenetic lineage closely related to *R. etli* CFN42 and *R. aethiopicum* HBR26. On the other hand, NAE182 is related to *R. acidisoli* FH23 and *R. fabae* lineage with 98% bootstrap support. All included strains were shown to be genetically distinct, with the exception of the Kenyan *Rhizobium tropici* strain NAK91 which turned out to have 100% sequence identity with *R. tropici* CIAT899 across all four loci.

Previous genetic interaction studies revealed preferential nodule occupancy of bean genotypes of Andean and Mesoamerican origin with of *R. etli* strains that had alleles at the *nodC gene* that were typical for each respective geography (20). We therefore inspected the phylogeny of this gene to allow analysis of the relation between *nodC* type and effectiveness or G_L_ x G_R_ interaction (Fig. 1b) and to ensure that selected local strains grouped with type strains known to nodulate bean. The local and type strains fell into five monophyletic clusters (Fig. 1b). In the first cluster the local strain NAE136 (originated from Ethiopia) grouped together with the *R. aethiopicum* type strain previously isolated from common bean in Ethiopia. The other Ethiopian *Rhizobium* sp. strain NAE182 formed a cluster with the reference strains *R. phaseoli* Brasil5 and, with a relatively low bootstrap value, *R. acidisoli* FH23. The third clade contained *R. etli* CFN42 and *R. phaseoli* ATCC14482. The Kenyan strain NAK91 was found to also be 100% identical to *R. tropici* CIAT899 for *nodC,* together forming a tight monophyletic clade with 100% bootstrap support that also included *R. freirei* PRF81 and *R. lusitanum* P1-7. The nodC gene of *R. multihospitium* formed a clade with those of the type strains of *R. pisi* and *R. fabae,* and none of these strains can nodulate bean effectively. As expected, the phylogenies of symbiotic and housekeeping genes show several incongruences (31, 99–102). Overall, the two phylogenies show that the selected provide good coverage of the genetic diversity among rhizobia.

### Occurrence of G_L_ x G_R_ interaction

**Fig. 2:**
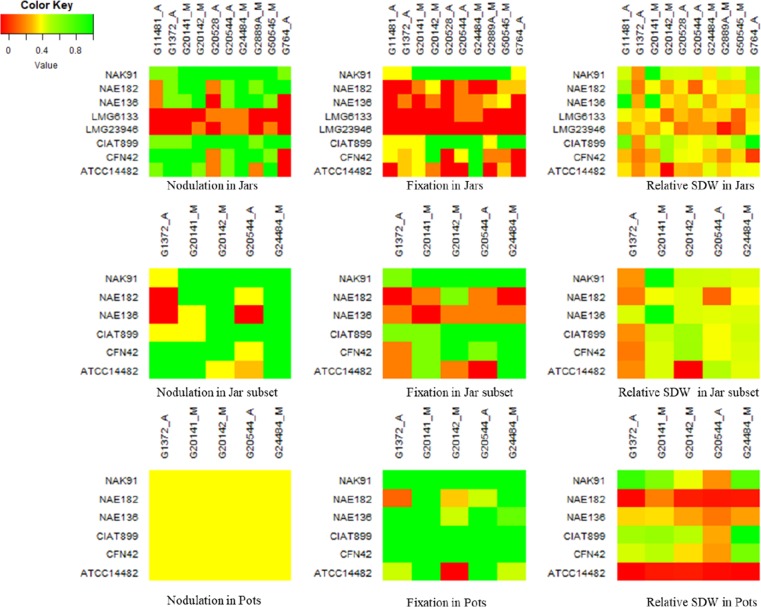
Nodulation, fixation and plant growth patterns of G_L_ x G_R_ interaction in jars and pots. Colour key was adjusted based on minimum, mean and maximum scores of nodulation, fixation and relative shoot dry matter for each case

Nodulation (nod+/-) and fixation (fix+/-) was scored on all strain by genotype combinations in the Leonard jar experiment, except for the controls (Fig 2). Informativeness of nodulation and fixation phenotypes for symbiotic effectiveness was confirmed by significantly higher relative shoot biomass in nod+ compared to nod-phenotypes and in nod+fix+ compared to nod+fix- phenotypes (Table S2). Six strains induced consistent nodulation on most genotypes, but occasionally failed to nodulate genotypes G11481, G20528, G2889A and G764, showing clear G_L_ x G_R_ for nodulation and fixation. Of the nodulating strains, only CIAT899 and NAK91 consistently formed pink-coloured nodules, suggesting these were Fix+. Combinations involving other strains frequently involved small nodules (Nod+) with white internal colour (Fix-), indicating ineffective N_2_ fixation. Such plant x rhizobium combinations were scored as Nod+/Fix-. Two strains, *R. multihospitium* LMG23946 and *S. meliloti* LMG6133, showed no - or very inconsistent nodulation across genotypes. Although the former groups together with CIAT899 in the housekeeping gene phylogeny, it falls into the same cluster as the latter for *nodC,* indicating that they share different Nod genes from CIAT899 (Fig. 1).

Significant G_L_ by G_R_ was observed for relative shoot biomass in the Leonard jar experiment (p <0.01) (Table 4). The six effective strains CIAT899, NAK91, NAE136, NAE182, CFN42 and ATCC14482 had significantly (p<0.05) positive relative shoot biomass in combination with one or more bean genotypes. Only CIAT899 and NAK91 had significantly positive relative shoot biomass in combination with six genotypes or more. Strains CFN42 and ATCC14482 were only significant in combination with a single genotype, producing relative shoot biomass 0.36, only half the maximum value of 0.72 that was observed for CIAT899. Not surprisingly, the two poorly-nodulating strains showed no evidence of being effective in combination with any of the plant genotypes, except strain LMG6133 induced fix+ nodules on only single plant out of five plants with genotypes G20544 and G24484.

**Table 4:** ANOVA table for G_L_ x G_R_ interaction in Leonard jar, subset of Leonard jars and pots

Growing conditions	Sources of variation	DF	Mean of squares				
			NN	NDW_(gm)_	RSDW	Ndfa_(gm)_	RNdfa
	G_L_	9	2837.7***	0.009***	0.28***	-	-
Jar full set	G_R_	7	12851.1***	0.015***	0.58***	-	-
	G_L_ x G_R_	63	860.2**	0.0017	0.12**	-	-
	G_L_	4	4864.1***	0.017**	0.420***	-	-
Jar subset	G_R_	5	4304.5***	0.004	0.17*	-	-
	G_L_ x G_R_	20	892.3	0.003	0.17***	-	-
	G_L_	4	3391.2***	0.04*	0.24***	2.5***	0.27**
Pot	G_R_	5	3060.3***	0.07***	117***	9.7***	1.40***
	G_R_ X G_R_	20	769.7	0.012	0.05	0.6**	0.08

G_r_ = genotypes; G_r_ = strains; Res = residuals; DF = degree of freedom; NN = nodule number; NDW = nodule dry weight; RSDW = relative shoot dry weight of the plant; Ndfa = estimated amount of nitrogen derived from atmosphere; RNdfa = relative Ndfa; gm = gram; “***” = significant at p < 0.001; “**” = significant at p < 0.01; “*” = significant at p < 0.05; “.” = significant at p < 0.1

**Fig. 3:**
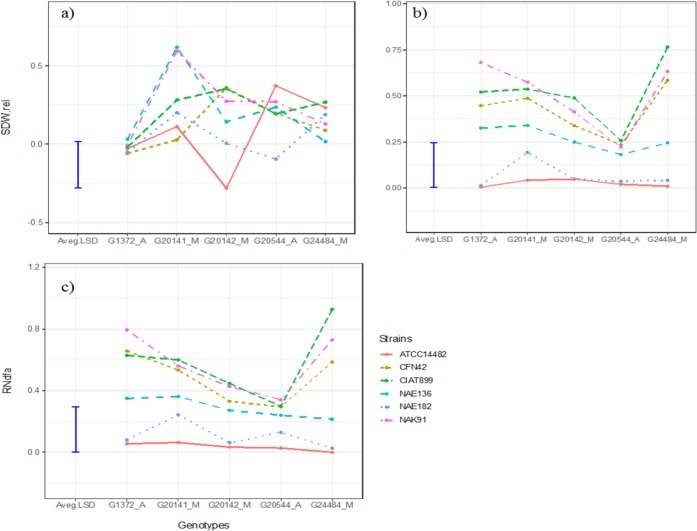
G_L_ x G_R_ interaction in beans in jars and pots. a) relative shoot dry weight (SDW.rel, in grams) in jars in grams; b) SDW.rel in pot; c) RNdfa is relative amount of nitrogen derived from atmosphere

Joint analysis of shoot biomass for the subset of genotypes and strains shared between the jar and pot experiments showed significant (p < 0.01) three-way interaction between type of experiment, G_L_ and G_R_ (Table S1). This reflected the fact that G_L_ x G_R_ interaction was significant in the jar experiment (p < 0.001, Table 4) while it was much weaker and not significant in pots (Table 4, Fig. 3). This suggests the result of effectiveness may not be applicable across growing conditions. However, in the pot experiment, G_L_ x G_R_ interaction was found to be significant (p < 0.0043) for estimated fixed N_2_ but not significant for relative amount of fixed N_2_ (Table 4), which was estimated based on the modified RSE calculation (see methods section).

**Table 5:** Effects of genepools, growth habit and country of origin on G_L_ x G_R_ interaction

	Sources	Response	DF	Mean squares		
				Jar	Pot	Pot
				RSDW	RSDW	Ndfa
Full set		GP	1	0.013	-	-
		G_R_	7	0.282*	-	-
	GP	G_P_ x G_R_	7	0.035	-	-
		GH	2	0.015	-	-
	GH	G_R_	8	0.282*	-	-
		G_P_ x G_R_	16	0.144	-	-
		GO	1	0.680*	-	-
	GO	G_R_	7	0.429**	-	-
		GO x G_R_	7	0.153	-	-

Where: GP = genepool; GH = growth habit; GO = genotype origin; Gr = strains; RSDW = relative shoot dry weight; Ndfa = nitrogen derived from atmosphere; = significant at p < 0.001; “**” = significant at p < 0.01; “*” = significant at p < 0.05; “.” = significant at p < 0.1

### Patterns of G_L_ x G_R_ interaction

**Fig. 4:**
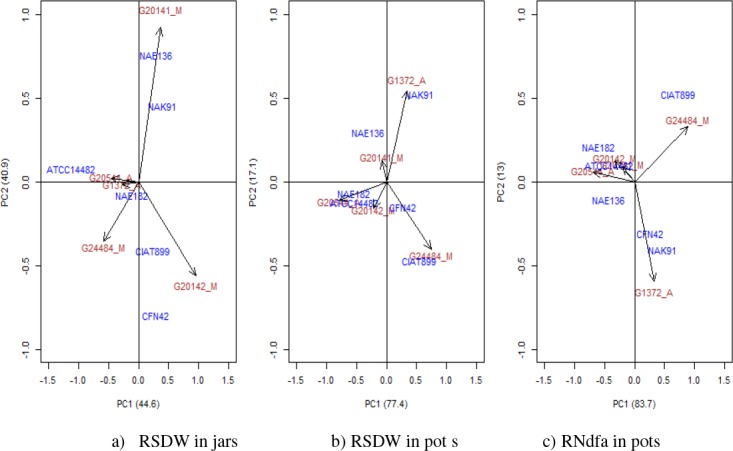
AMMI biplots: RSDW is relative shoot dry weight and RNdfa is relative amount of N_2_ derived from atmosphere. Bean genotypes are indicated by red colour to which black arrows are pointing while the Rhizobium strains are indicated by blue colour

The G_L_ x G_R_ interaction in the Leonard jar experiment, though highly significant, was mainly driven by two strains, *R. etli* CFN42 and *Rhizobium* sp. NAE136 (Fig. 4a; Fig. S4). Removing these strains led to a loss of significance for the interaction (p = 0.38). Similarly, four genotypes (G764, G11481, G20141 and G20142) showed particularly high variance in effectiveness (Fig. S4), their removal also leading to loss of significance (p = 0.56). We found no significant interaction between strain and either genepool, growth habit or country of origin, either in the full and reduced set of combinations of G_L_ x G_R_ (Table 5; Fig. S3).

**Table 6:** AMMI decomposition of genotype and strain main effects in jars and posts experiments.

Growing conditions	Sources	DF	Mean of squares			
			NN	NDW_(gm)_	RSDW	Ndfa_(gm)_
	PC1	15	1.18*	1.55*	1.46***	-
	PC2	13	1.28*	0.66	1.52***	-
	PC3	11	1.36*	0.52	1.44**	-
Jar full set	PC4	9	0.85	0.39	0.87	-
	PC5	7	0.58	0.33	0.67	-
	PC6	5	0.53	0.04	0.61	-
	PC7	3	0.04	0.02	0.10	-
	PCA1	8	1.09	1.05	1.73**	-
Jar joint	PCA2	6	1.11	0.31	2.11***	-
	PCA3	4	0.95	0.16	0.99	-
	PCA4	2	0.21	0.17	0.25	
	PCA1	8	1.29.	1.30	1.12**	189***
Pot joint	PCA2	6	1.27.	0.44	0.33	0.10
	PCA3	4	0.43	0.53	0.14	0.10
	PCA4	2	0.45	0.02	0.05	0.07

Where, NN is nodule number, NDW is nodule dry weight, RSDW is relative shoot dry weight and Ndfa is nitrogen derived from atmosphere; gm = gram; “***” = significant at p < 0.001; “**” = significant at p < 0.01; “*” = significant at p < 0.05; “.” = significant at p < 0.1

Closer inspection of the results for the subset of genotypes and strains evaluated in the two experiments (Fig. 3; Fig. 4) revealed a strong G_L_ x G_R_ interaction in the jar experiment. In this subset the interaction was again driven mostly by strains CFN42 and NAE136 which interacted positively with genotypes G20142, G20141 and to a lesser extent by strains ATCC14482 which interacted negatively with genotype G20142. These interactions were associated with two significant axes in the AMMI analysis (Table 6).

As mentioned above, G_L_ x G_R_ was much weaker in the pot experiment and no substantial crossover interactions were observed for either relative shoot biomass or fixed N_2_ (Fig. 3). Strains CIAT899 and NAK91 were the best performing strains across all genotypes, followed by strains CFN42 and NAE136. The performance of strains ATCC14482 and NAE182 was consistently poor for both criteria with only the latter showing some evidence of effectiveness on genotype G20141 (p = 0.026). In accordance with this weak G_L_ x G_R_ interaction, only a single significant axis (PCA1) was identified in the AMMI analysis (Table 6). Along this axis, the strong positive interactions of strain CIAT899 with genotype G24484 and strain NAK91 with genotype G1372 dominate the interaction for relative shoot biomass (Fig. 4b). With regard to relative Ndfa, strains CIAT899 and NAK91 were stable across the genotypes while the poorly fixing strains (ATCC14482 and NAE182) accounted for much of the variations (Fig. 4c). Removing either of these increases the p-value for the interaction term for relative Ndfa to 0.38 or 0.21 respectively.

As described above, molecular analysis showed that symbiotic gene *nodC* phylogeny clustered together strain *R. etli* CFN42 with strain *R. phaseoli* ATCC14482 (Fig. 1). These strains induced quite different patterns of nodulation, fixation and shoot biomass across the bean genotypes in both growth environments (Fig. 2). Similarly, strains *R. phaseoli* ATCC14482 and *Rhizobium sp.* NAE182 were different in symbiotic gene relationship but they appeared to have relatively similar symbiotic performance across the bean genotypes (more explainable in pot experiment). On the other hand, a high genetic identity (both in symbiotic and housekeeping genes) between *R. tropici* strains CIAT899 and NAK91 revealed similar patterns of nodulation and symbiotic effectiveness. The remaining strains appeared to show inconsistent patterns of symbiotic effectiveness but have occupied different positions in *nodC* phylogeny.

## Discussion

Beans are generally regarded to have poor N_2_ fixation potential compared to other legumes (5–7) but their symbiotic effectiveness is known to vary with legume genotypes, rhizobial strains (13, 74) and their combinations. The occurrence of genotype by strain interaction is therefore of great relevance, since it may represent either opportunities or challenges for enhancing N_2_ fixation. The prospect of identifying predictable patterns in G_L_ x G_R_, in terms of genetic identity of germplasm or strains, is particularly enticing as it would open the possibility of targeting different variety types with superior rhizobial strains. The observation of preferential nodule occupancy by *R. etli* types of the same geographic origin as the co-infected bean varieties (20) suggests that such patterns may indeed exist. Although prior studies have found the interaction in beans in terms of both nodulation (75, 76) and effectiveness (13, 22, 73), our study screens a wide set of genetically diverse cultivars and selects taxonomically distinct rhizobium strains with the aim of evaluating both the occurrence and patterns of G_L_ x G_R_ in bean.

Our results confirm that there is indeed G_L_ x G_R_ interaction in beans. In terms of nodule formation, four genotypes and four strains were involved in unsuccessful combinations with generally well performing symbiotic partners. Genotype G764 for example, failed to form nodules with three consistently nodulating strains, among which is the highly effective *R. etli* strain CFN42. Differential nodule formation is perhaps the most classic expression of G_L_ x G_R_ interaction, first observed in pea (103) but later reported in other species such as faba bean, vetch (104–106) and, using different slow-growing *Bradyrhizobium* and fast-growing *R. fredii* strains, in bean (107). Nodule formation is a key symbiotic trait that is determined by genetic factors of both host and symbiont (23, 63), with host gene sym2 and rhizobium gene nodX specifically identified as underlying the G_L_ x G_R_ interaction in soybean (106) and peas (108, 109).

We also observed the occurrence of strains that, although they caused consistent nodulation, presented inconsistent fix+/- phenotypes across the different bean genotypes, suggesting that infection does not always translate into a successful symbiosis. Fix+/-phenotypes were more prevalent for less effective strains *Rhizobium* sp. NAE182 and *R. phaseoli* ATCC14482 and the moderately effective strain *Rhizobium* sp. NAE136. Additionally, the significantly higher biomass observed in Fix+ phenotypes suggests that it might be a useful measure of effectiveness (Fig. 2, Table S2). Still, cytological and molecular studies in legumes have confirmed that specific legume strain combinations may fail to fix N_2_ after nodulation (77, 78, 108), possibly due to strain specific differences in nod factor decorating genes (110, 111) and incompatibility between the interacting symbiotic partners (112). A study on a promiscuous non-legume *Parasponia andersonii* also reported failed symbiosis caused by the infecting *R. tropici* strain that become embedded in a dense nodule matrix but remained viable without fixing atmospheric nitrogen (112).

Fixation of atmospheric N_2_ and the associated accumulation of plant biomass are the most direct measures of symbiotic effectiveness. The evidence for G_L_ x G_R_ interaction for these traits in agricultural legumes such as Bambara groundnut (67), lentil (68), pea (69), soybean (70, 113), lotus (114), white clover (72), peanut (71), Medicago (23, 39, 115), and wild species such as *Acacia* spp. (74, 116) attest the marked variability of host genotypes to establish effective symbiosis with specific strains. In bean, results on G_L_ x G_R_ interaction for fixation and biomass have been mixed, with some studies reporting significant interaction for these traits (21, 73) while others have reported lack of interaction (12). Here we find strong and significant G_L_ x G_R_ for relative shoot biomass in our Jar experiment, but this interaction largely disappears in a follow-up experiment using pots, with only a single significant AMMI component, due to the weak positive interaction of one genotype with CIAT899, remaining as evidence for interaction.

This differential outcome is striking, since in the jar experiment the most effective strains such as CIAT899, NAK91 and CFN42 were completely ineffective with one or more of the bean genotypes while their performance was stable in the pot experiment. Environmental effects on patterns of G_L_ x G_R_ interaction have been reported in the literature. A study in Medicago (66) revealed changes in specific interactions as a function of nitrate, while in bean a study on two Tunisian soils revealed significant inoculation treatment x cultivar x soil interaction (25), which is congruent with the experiment x cultivar x strain interaction that we observe. In principle, any environmental factor affecting either bacterial growth and survival or plant vigour (reviewed in Zahran (117)) may affect symbiotic effectiveness and G_L_ x G_R_ interaction. Soil pH for example, was shown to significantly influence the performance of *R. tropici* strains in bean (30), as well as competition between strains (118). Availability of plant nutrients such as nitrogen and phosphate (23, 119) are also relevant for the outcome of symbiosis. In our case, media in both experiments were standardized for nutrient and initial pH adjustments (although pH may admittedly change through time) suggesting that other limiting factors, such as the marked difference in rooting volume and the associated differences in biomass, differentially affected the ability of certain bean genotypes to benefit from the symbiosis with specific strains. Whatever the underlying causes, the differential outcome between our two experiments does implicate limiting growth conditions as a factor enhancing G_L_ x G_R_. The fact that among the bean studies mentioned above the one with the strongest evidence for G_L_ x G_R_ (73) was performed in plastic tubes, while the one reporting a lack of interaction (12) used pots, would seem to support this notion. Pot experiments thus seem to have more potential for finding G_L_ x G_R_ patterns of interest that may be repeatable under practical conditions, although only field testing will determine to what extent this is the case.

In so far as the G_L_ x G_R_ interaction observed in the jar experiment is biologically meaningful, our results show no differences in effectiveness or interaction due to bean genepool, growth habit and breeding history nor did we find any evidence that certain nodC or multi-locus clades are predictive of symbiotic outcome. This finding is in line with evidence from studies in other legumes that suggest that symbiotic interactions may not typically show larger geographic or intraspecific phylogenetic patterns, but rather that they are specific at the level of individual genotypes (or close genetic relatives) and strains (55) and independent of the geographic origin of host or symbiont (53). Because populations are genetically differentiated groups, the outcomes of their interspecific interactions (traits) differ in their geographic ranges (120). On the other hand, a recent study across several native Australian legume species and rhizobium strains from different phylogroups found evidence of host-symbiont sympatry in some species as well as significant host plant time rhizobium phylotype interaction, suggesting that some geographic or taxonomic co-evolution may occur (74). In bean, a study screening 820 genotypes for response to a single *R. phaseoli* strain (KIM5) found that among the 50 most extreme nodulation phenotypes, the poor nodulating genotypes were almost exclusively from Mesoamerica (Rosas et al. cited in (106)), suggesting a role for intraspecific genetic structure in symbiotic compatibility, while the aforementioned study by Aguilar et al. (20), clearly suggests that a preference for sympatric rhizobia within the two bean genepools. Our results suggest that targeting specific types of germplasm to specific types of strains is not likely to be feasible, although admittedly they were obtained on sterile medium in the absence of competition which may play a large role in actual soils. In this respect, it will probably be more rewarding to identify strains that are better adapted to different locations or soils, for which the literature seems to provide some evidence (25, 30, 56, 119, 121, 122).

What our study does demonstrate, is that there are strong main effects of genotype and strain on symbiotic effectiveness. This result is similar to that observed in bean (13, 21) and in other legumes (23, 33, 114, 123, 124) but contrasts with results reported by Buttery et al. (12), where no differences in either bean genotypes or strains were found. The observation that individual bean varieties differ markedly in their ability to benefit from effective symbiosis suggests that breeding bean varieties for enhanced N_2_ fixation is possible, although further field experimentation would need to confirm this. With respect to the former, the strong and consistent performance of the commercial inoculant *R. tropici* CIAT899 and the identical local strain NAK91 are encouraging, as demonstrate that good inoculants that work across different types of germplasm are available. It also shows that newly isolated candidates have relatively weak performance compared to elite strains, as illustrated by NAE182 and NAE136. A potential exception could be the Kenyan strain NAK91, which had equal performance to CIAT899 and CFN42. The observation that this strain was 100% genetically identical at both *nodC* and concatenated housekeeping genes, however, suggests the possibility that NAK91 is actually the same strain as CIAT899, which has been used as inoculant in Africa and could have persisted in the soil. Other studies focusing on bean rhizobia in Kenya showed similar cases. For instance, strains isolated in acid Daka-ini soil (pH 4.5) were symbiotically equally or more effective than *R. tropici* CIAT899 and had pronounced similarity in restriction fragment fingerprint (30). Recently, Mwenda et al. (32) who isolated NAK91, reported other strains induced comparable biomass to CIAT899, although this suggests that *R. tropici* strains might have adapted well in acid soils of Kenya. We did observe slight differences in specific effectiveness between the two strains and further sequencing may still prove that NAK91 is genetically different from CIAT899, as was reported for the *R. tropici* strain WUR1 which was shown by full genome sequencing to differ from CIAT899 at a number of nucleotide positions.

In conclusion, although our initial jar experiment detected strong G_L_ x G_R_ interaction in beans in terms of both nodulating and effectiveness, this interaction was due to individual genotypes and strains without showing any genetic or taxonomic pattern. Our confirmatory experiment using larger growing volume removed most evidence of interaction and confirmed the stable superiority of the well-known strains CIAT899 and CFN42 and the local strain NAK91 while revealing differences in N_2_ fixation and biomass accumulation between specific genotypes. Thus, the stable performance of these strains should be further evaluated in multi-locational field trials. The possibility that growth conditions used for the experiments influenced the occurrence and patterns of symbiotic interaction provides a cautionary message to consider in future studies and suggests that follow-up field trials are to be recommended.
