# Supplementary material for: Genetic Interaction Studies Reveal Superior Performance of Rhizobium tropici CIAT899 on a Range of Diverse East African Common Bean (Phaseolus vulgaris L.) Genotypes
Source: Appl Environ Microbiol. 2019 Nov 27;85(24):e01763-19. doi: 10.1128/AEM.01763-19 (PMC6881787; doi:10.1128/AEM.01763-19)
Supplement: Supplementary file 1 [file AEM.01763-19-s0001.pdf]

Table S1: ANOVA table for joint experimental data

Sources	DF	Mean squares		
		NN	$NDW_{(gm)}$	RSDW
Exp	1	22545.4***	0.459***	0.240.
G_L	4	1035.1	0.040**	0.319***
G_R	4	6712.9***	0.044***	1.021***
Exp x G_L	4	7226.1**	0.013	0.342***
Exp x G_R	4	646.0	0.034**	0.302***
G_L x G_R	16	920.2.	0.007	0.091*
Exp x G_L x G_R	16	741.0	0.007	0.108**

Where Exp = experiments; G_L = legume genotypes; G_R = *Rhizobium* genotypes; DF = degree of freedom; NN = nodule number; NDW = nodule dry weight; RSDW = relative shoot dry weight of the plant; gm = gram; "***" = significant at $p < 0.001$; "**" = significant at $p < 0.01$; "*" = significant at $p < 0.05$; "." = significant at $p < 0.1$

Table S2: Effects of nodulation and fixation on plant biomass

Source		Num-DF	SDW.rel		
			MS.	F-value	Pr (>F)
Jar	Nod+/-	1	1.4982	17.816	0.0000354***
Jar	Fix+/-	1	0.3446	4.0552	0.04558 *
Pot	Fix+/-	1	0.37099	10.245	0.001678 **

Where: SDW.rel represents relative shoot dry weight; Jar indicates experiment conducted in Jar; Pot indicates experiment conducted in pot; Nod+/- stands for presence or absence of nodules; and Fix+/- indicates whether there is nitrogen fixation or not

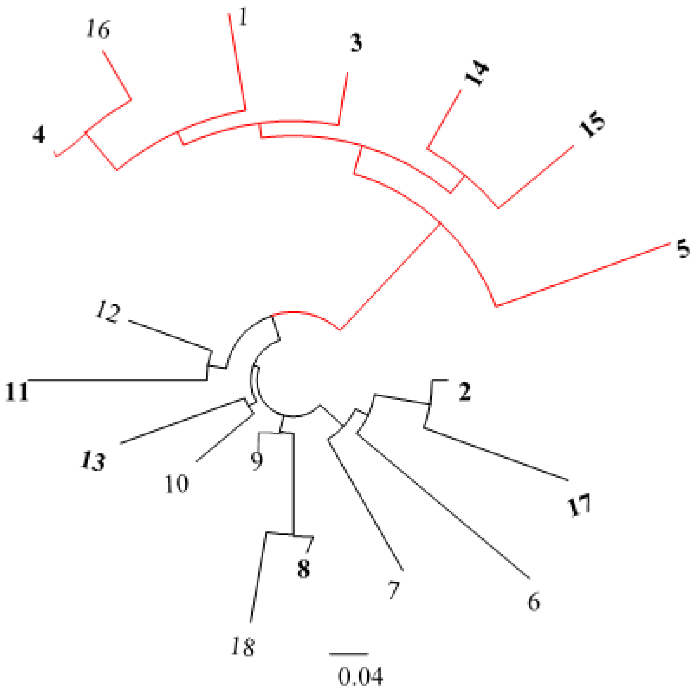

Fig. S1: Neighbour joining tree of bean genotypes, reconstructed from Asfaw et al. [2] based on pairwise genetic distances. Red coloured branch of the tree represents Andean genepools while the black represents the Mesoamerican genepools. The numbers represent cluster groups that contain individual genotypes.

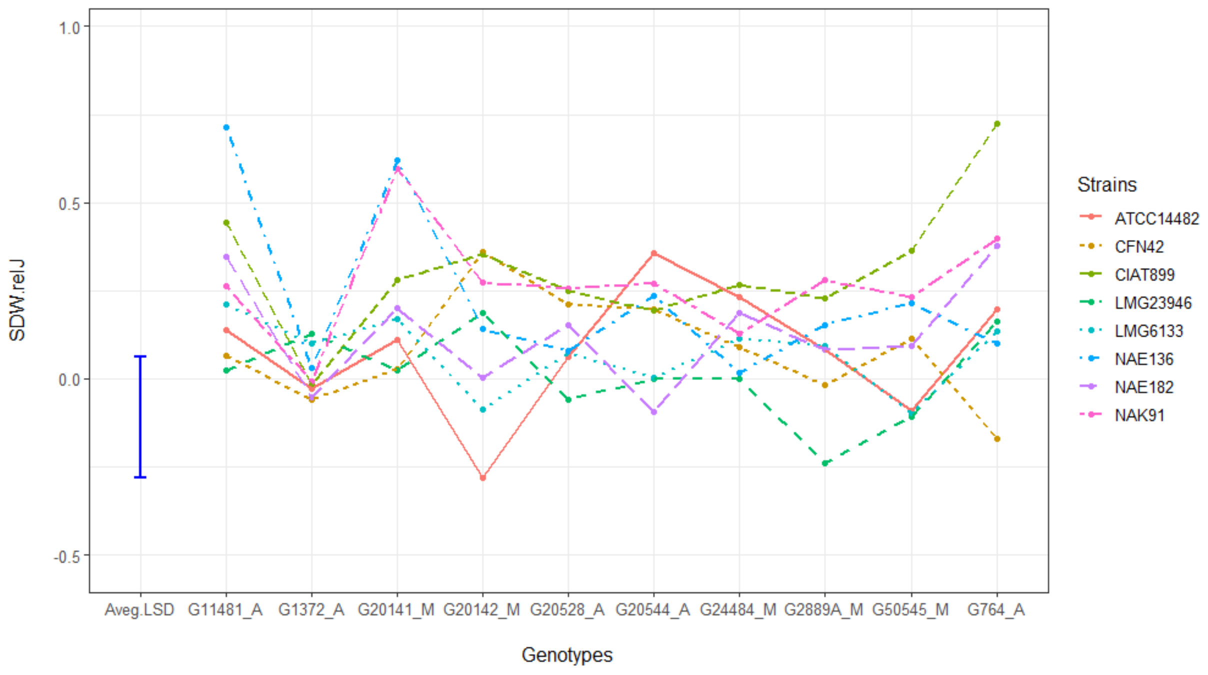

Fig. S2: Predict means plot of bean genotypes x *Rhizobium* strains interaction. Analysis of a full data set in jar experiment; positive and negative controls were excluded. Where, SDW.relJ represents relative shoot dry weight (in grams) of the jar experiment

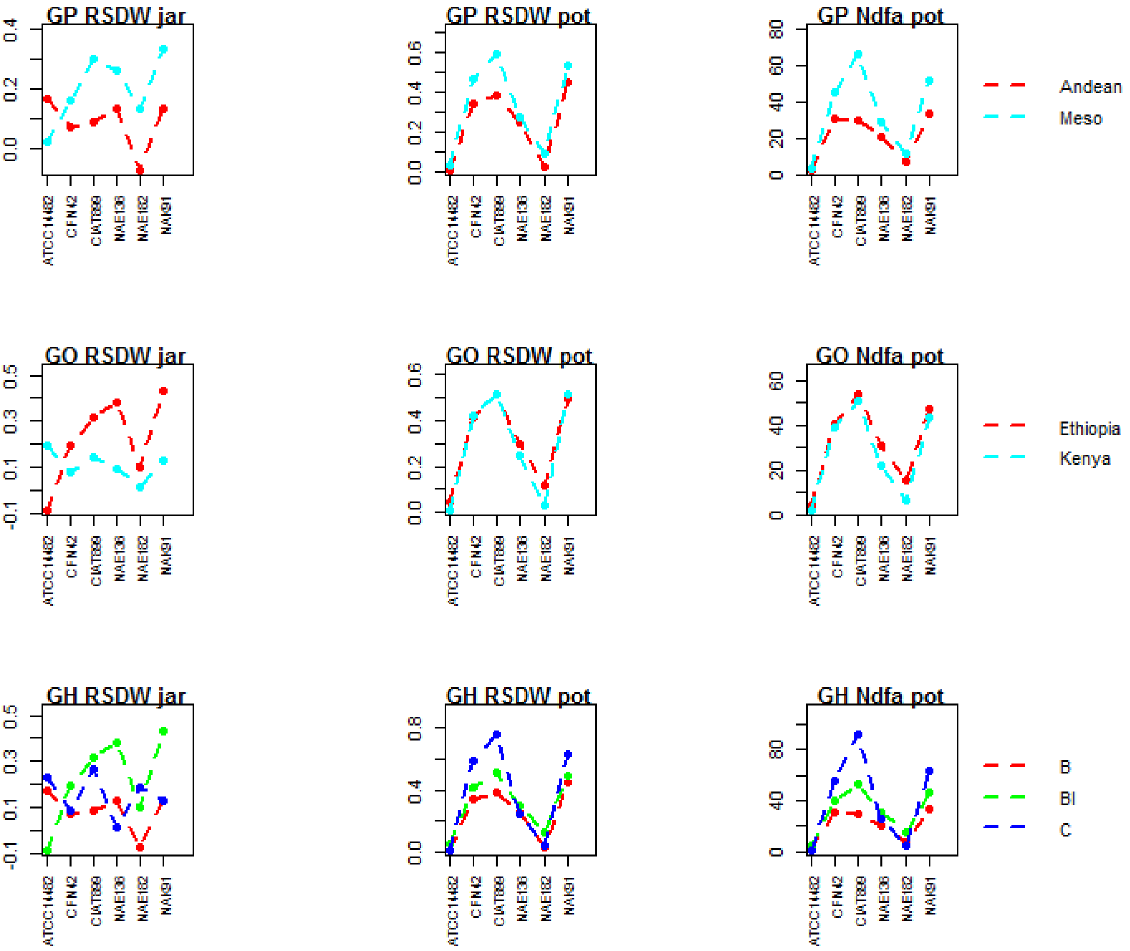

Fig. S3: Effects of genepools, growth habit and country of origin on G_L x G_R interaction. Where, GP = genepools, GH = growth habits, GO = genotype country of origin (history of breeding), RSDW = relative shoot dry weight (in grams), Ndfa = Nitrogen derived from atmosphere (in percentage), Meso = Mesoamerican genotypes, Andean = Andean genotypes, B = bush type beans, BI = bush indeterminate type beans and C = climbing type beans

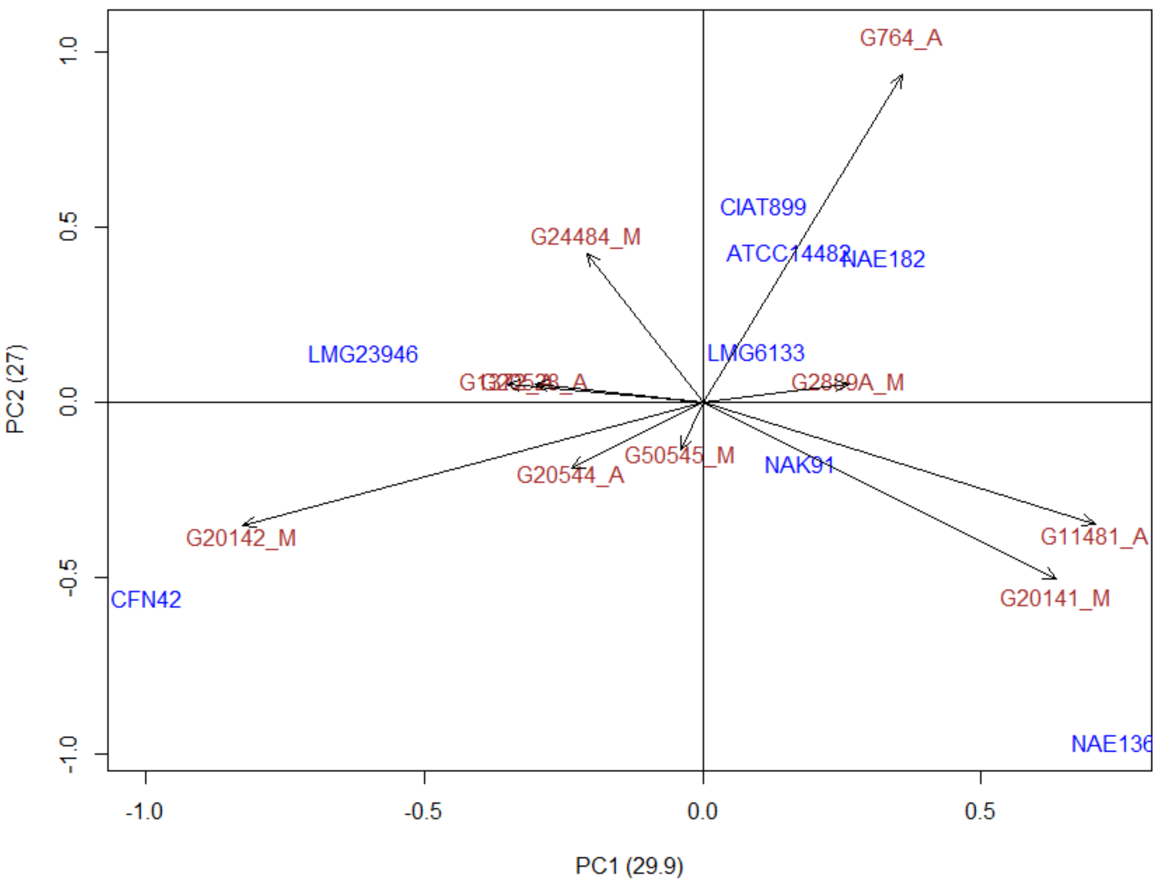

Fig. S4: AMMI biplot using full data from jar experiment. Bean genotypes represented by red names (in terms of relative shoot dry weight (RSDW) to which black arrows are pointing and the blue names represent rhizobia used to inoculate the bean genotypes. Bean genotypes and rhizobium strains that are far away from the origin (length of the arrow) contribute the majority of variations in RSDW. The closer a genotype to a given strain, the higher correlation exists between them and this represents best matching combinations. Strains and genotypes that are closer to the origin of the biplot are the stable ones.
